# Maternal immune activation does not affect maternal microchimeric cells

**DOI:** 10.1242/bio.061830

**Published:** 2024-12-23

**Authors:** Alexandria Borges, Naoki Irie

**Affiliations:** ^1^ Graduate School of Science, Department of Biological Sciences, The University of Tokyo, 113-0033 7-3-1 Bunkyo-ku, Hongo, Tokyo, Japan; ^2^ Research Center for Integrative Evolutionary Science, SOKENDAI 240-0193 Shonan Village, Hayama, Kanagawa, Japan

**Keywords:** Maternal microchimerism, Immunology, Embryonic development, Cell-level epigenetics

## Abstract

We are naturally chimeras. Apart from our own cells originating from the fertilized egg, placental mammals receive small numbers of maternal cells called maternal microchimerism (MMc) that persist throughout one's whole life. Not only are varying frequencies of MMc cells reported in seemingly contradicting phenomena, including immune tolerance and possible contribution to autoimmune-like disease, but frequencies are observable even among healthy littermates showing varying MMc frequencies and cell type repertoire. These varying differences in MMc frequencies or cell types could be contributing to the diverse phenomena related to MMc. However, factors biasing these MMc differences remain largely unknown. Here, we tested whether immunological activation leads to differing MMc frequencies, based on our recent study that suggests that most maternal cells are immune-related. Unexpectedly, fluorescence-activated cell sorting analysis on the murine spleen, thymus, and liver following maternal immune activation by mid-gestational lipopolysaccharide intraperitoneal injections detected no significant difference in the number, or ratio of, immune-related maternal cells in the tested embryonic organs of healthy offspring. These findings suggest that MMc frequencies remain stable even under immune-activated conditions, implying a possible control system of MMc migration against changes in the immunological conditions.

## Teaser

**Summary:** Maternal microchimerism (MMc) varies in our bodies through unknown factors. We tested whether maternal immune activation alters its proportion, but no significant differences were detected, thus suggesting an MMc control system.

## INTRODUCTION

*From birth, our mothers never truly leave us. We have maternal cells throughout our bodies that last our whole lives.*

Within an organism of placental mammals exists a mixture of themselves and non-self cells from their mother. Maternal microchimerism (MMc) is the phenomenon where a low level of maternal cells exists in progeny, entering through the placenta during embryogenesis and via breastmilk after birth (Lo et al., 1996; Gammill and Nelson, 2010; Aydın et al., 2018). These rare cells are typically prevalent at a low frequency of around 1 in 100,000-10,000,000 cells (Mold et al., 2008; Dutta et al., 2009; Bakkour et al., 2014; Castellan and Irie, 2022) and have been observed in various fetal tissues such as the lungs, spleen, liver, and skin (Jonsson et al., 2008; Nelson, 2012). Recently, maternal cells have been suggested to be primarily immune-related (Fujimoto et al., 2022), along with previous findings of maternal cell types including T cells, B cells, macrophages, and dendritic cells (Loubière et al., 2006), as well as tissue-related like keratinocytes, cardiac myocytes, and neuron-like cells (Stevens et al., 2003, 2009; Snethen et al., 2020). Concurrent with its prevalence in various locations, differing frequencies of maternal cells are also associated with contrasting conditions. MMc is primarily highlighted for its role in promoting fetal tolerance against non-inherited maternal antigens (NIMA) (Dutta and Burlingham, 2011; Kinder et al., 2015; Mishra et al., 2021), with recent findings suggesting that maternal cells may have the potential to suppress cytotoxic responses in progeny and regulate their neonatal immune reactivity (Castellan and Irie, 2022). Another potential role is tissue regeneration, exemplified in the pancreas of Type I diabetics (Nelson et al., 2007). In contrast, higher frequencies of MMc have been reported in auto-immune and auto-immune-like diseases such as juvenile dermatomyositis (Reed et al., 2004), systemic sclerosis (Lambert et al., 2004), auto-immune diabetes (Roy et al., 2011), and biliary atresia (Muraji et al., 2008, 2009; Irie et al., 2009), implying possible contributions to the aggravation or even onset of these diseases. While the increase of MMc frequencies could merely be a result of the mentioned various phenomena (e.g. inflammatory reactions), variations in maternal cell proportion and cell-type repertoire could also be the trigger or contributing factor for the various conditions. Consistent with this, patients with biliary atresia were reported to have higher MHC compatibility with their mothers (Irie et al., 2009). Although biasing factors contributing to differences in frequencies of MMc and cell type repertoire are largely unknown, immune capacity (Müller et al., 2001) and MHC compatibility between mother and progeny (Silveira et al., 1993; Balle et al., 2022) are reported to contribute to a higher frequency of MMc cells. Recent evidence has shown that additional factors may be at play as variations in frequency and cell types of MMc cells were found across siblings, supporting that differing MMc frequency readily exists even among healthy individuals with similar genetic backgrounds (Fujimoto et al., 2021, 2022). The embryonic maternal cell repertoire was found on average to be predominately immune-related (67%), consisting of mainly myeloid cells and granulocytes as well as terminally differentiated tissue-specific cells (25%), and stem/progenitor cells (8%) (Fujimoto et al., 2022). This observation of the maternal cell repertoire coincides with previous knowledge (Piotrowski & Croy, 1996; Loubière et al., 2006), and further implies that unknown biasing factors may include an immune component. Therefore, it was a possibility that an increase of immune cells following immune activation in the mother may potentially result in an influx of immune cells that may migrate towards offspring, with attempts to observe phenotypes with artificially increased MMc previously untested until now.

With this regard, maternal immune activation (MIA) is an attractive candidate. Maternal infection during embryogenesis has been linked to an abnormal immunity in offspring including immune overreaction and immune response failure and associated with potential epigenetic modifications and various immune-related disorders, such as Type 1 diabetes mellitus and allergic disease (Shimizu et al., 2023). Lipopolysaccharide (LPS) can initiate maternal immune activation, and injections into pregnant dams have shown evidence of causing injury to both progeny, and to dam placentas (Fricke et al., 2018). Therefore, we hypothesized that MIA of the pregnant dam through LPS injection might be a biasing factor that increases the proportion of MMc in progeny.

## RESULTS

### Maternal cell detection strategy utilizing GFP fluorescence

**Fig. 1.**
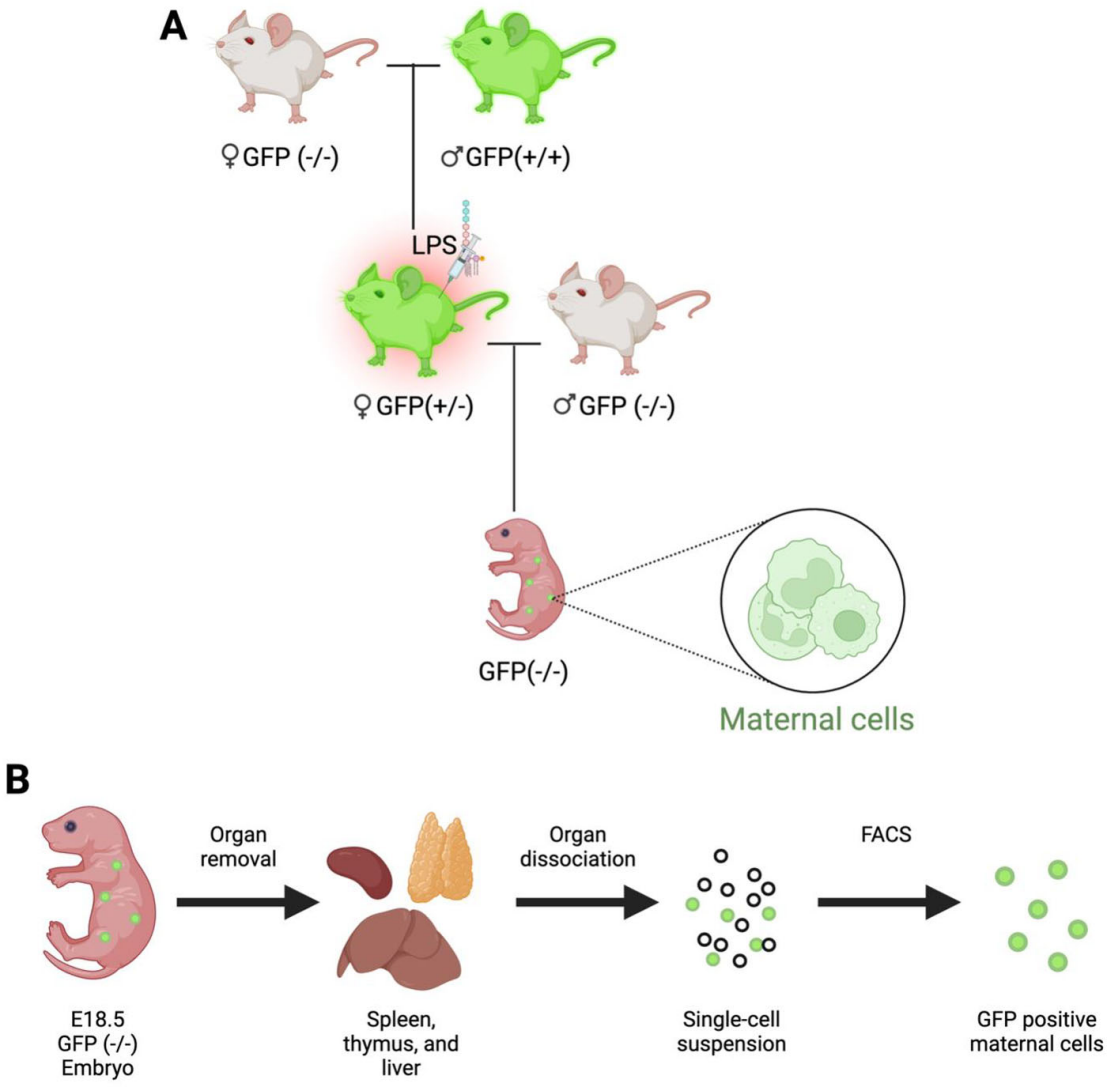
**Murine mating schematic for GFP-expressing maternal cell detection.** (A) Mating schematic to obtain GFP (−/−) embryos with GFP (+/−) maternal cells. (B) Experimental flow of maternal cell detection. Following the retrieval of E18.5 embryos, the spleen, thymus, and liver were dissected. The organs undergo dissociation, where the single-cell suspension is analyzed by FACS analysis for the isolation of GFP-positive maternal cells. The protocol was initially proposed and executed by Fujimoto et al. (2021, 2022). Figure made using BioRender.

In order to detect maternal cells in embryonic progeny, female GFP heterozygote transgenic mice were mated with GFP (−/−) males, where the pregnancy was confirmed by the presence of a copulation plug, marking E0.5. The pregnant dams received an LPS or PBS injection 11.5 days post conception (dpc). At E18.5, The GFP-negative offspring were retrieved through uterus dissection and were utilized for the observation of maternal cells (Fig. 1A). Following the retrieval of the GFP (−/−) embryos, embryos underwent removal of their spleen, thymus, and liver. The organs were processed for dissociation, single-cell suspension, and ultimately underwent FACS analysis for the estimation of maternal cells (Fig. 1B, Fig. S1).

### Embryos from LPS-injected dams had a developmentally impaired/fatal phenotype

**Fig. 2.**
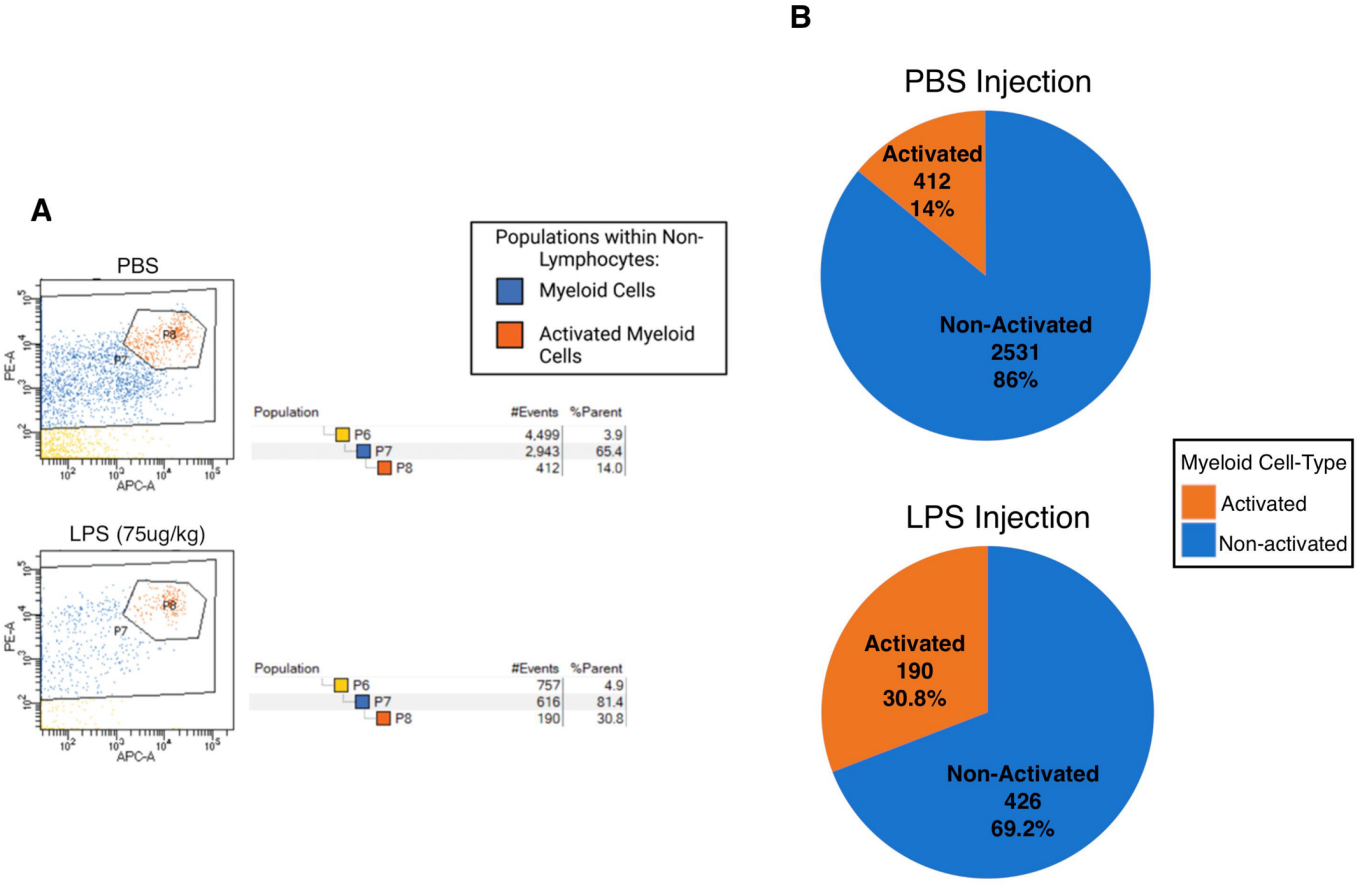
**75 µg/kg LPS dosage showed an increase in activated immune cells.** (A) FACS analysis of myeloid populations. Whole blood from WT B6, non-pregnant 10-week-old female injected with LPS at 75 µg/kg. The CD45(+)CD5(−)CD19(−) non-lymphocyte cell population (P6) was further gated for the Cd11b+ myeloid cell population (P7) and Ly6b.2-activated macrophages and inflammatory monocytes (P8). (B) Comparison of activated and non-activated myeloid cells**.** In PBS-injected dams, activated myeloid cells (*n*=412) comprised 14% of the total myeloid cell population (*n*=2943). In LPS-injected dams, activated myeloid cells (*n*=190) comprised 30.8% of the total myeloid cell population (*n*=616). *P*≤0.001. The *P*-value was obtained by performing Fisher's exact test.

**Fig. 3.**
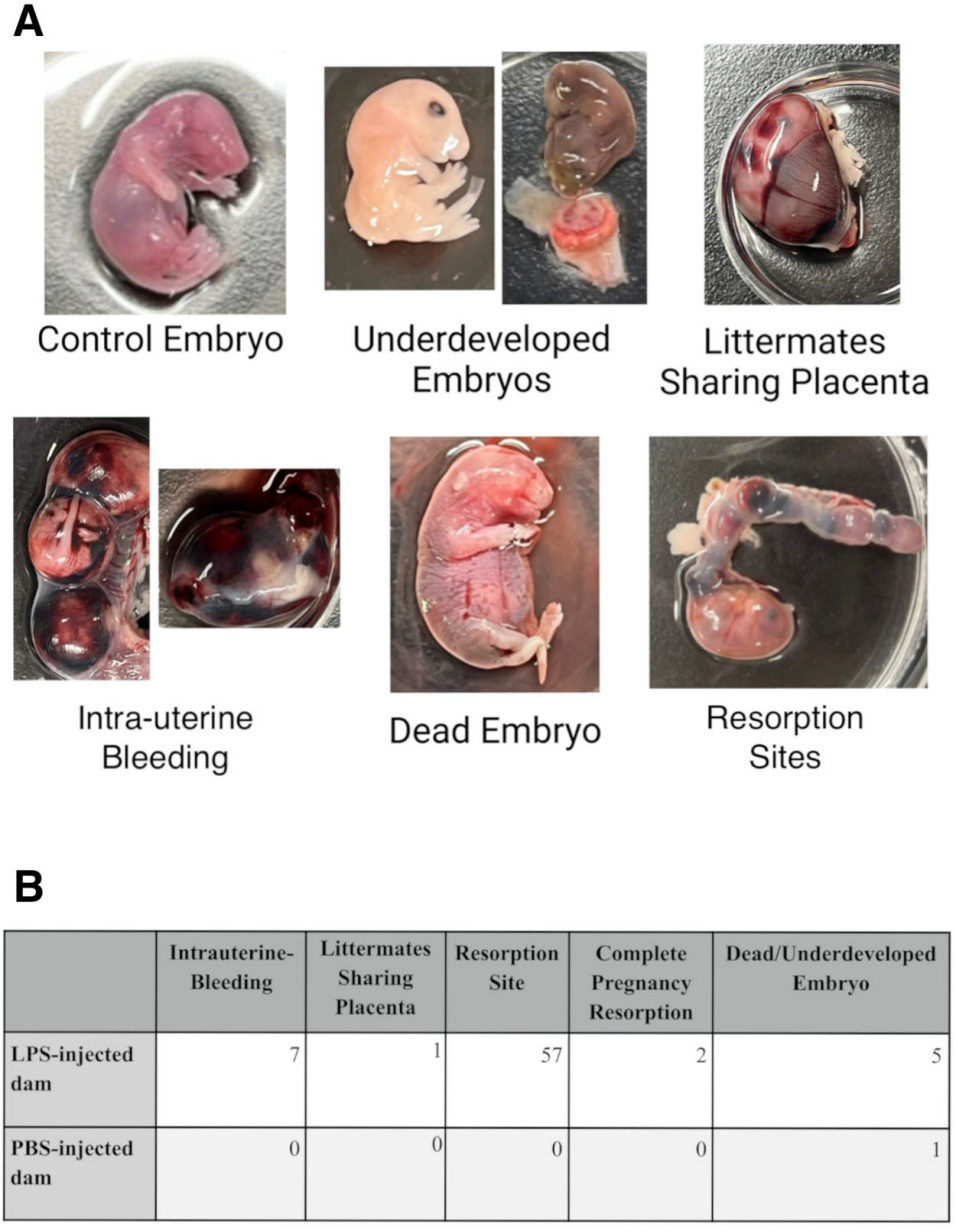
**Examples of anomalies found in the litters of LPS-injected dams.** (A) Anomalies were frequently found to affect both the embryo and placenta in the LPS-injected group, suggesting an immune activation response is taking place. No embryos of these kinds of phenotypes were used in the following analyses. (B) Occurrence of fetal/pregnancy anomalies in injected dams**.** Observation of anomalies following embryo retrieval at E18.5.LPS-injected dams *n*=16, PBS-injected dams *n*=8.

GFP heterozygote transgenic mice were intraperitoneally injected at mid-gestation (11.5 dpc) with LPS, a component of gram-negative bacteria, to initiate a maternal immune response. Injections were delivered at a dosage of 75 µg/kg, motivated by the work of other LPS-induced maternal inflammation murine experiments (Fricke et al., 2018). To determine if the dosage was satisfactory to implement an immune response change, we performed FACS analysis following LPS-injection or PBS-injection (Fig. 2A), where a higher proportion of activated myeloid cells were present in the LPS-injected group (Fig. 2B). Of the LPS-injected dams' litters, 13 out of 16 litters (>80%) had at least one developmentally impaired, dead, intra-uterine bleeding, resorption site, or complete pregnancy resorption (Fig. 3A) compared to only one occurrence in a PBS-injected litter (Fig. 3B), suggesting that LPS injections led to substantial immune activation, causing malformation to both progeny as well as to dam placentas. As analysis of the degraded deceased embryos was unobtainable (and may have been caused by non-microchimeric hemorrhage), GFP-negative, seemingly healthy littermates of embryos that shared the same intrauterine environment with the occasional abnormal phenotype were used in flow cytometry analysis.

### A difference in maternal cell proportion was not detectable

**Fig. 4.**
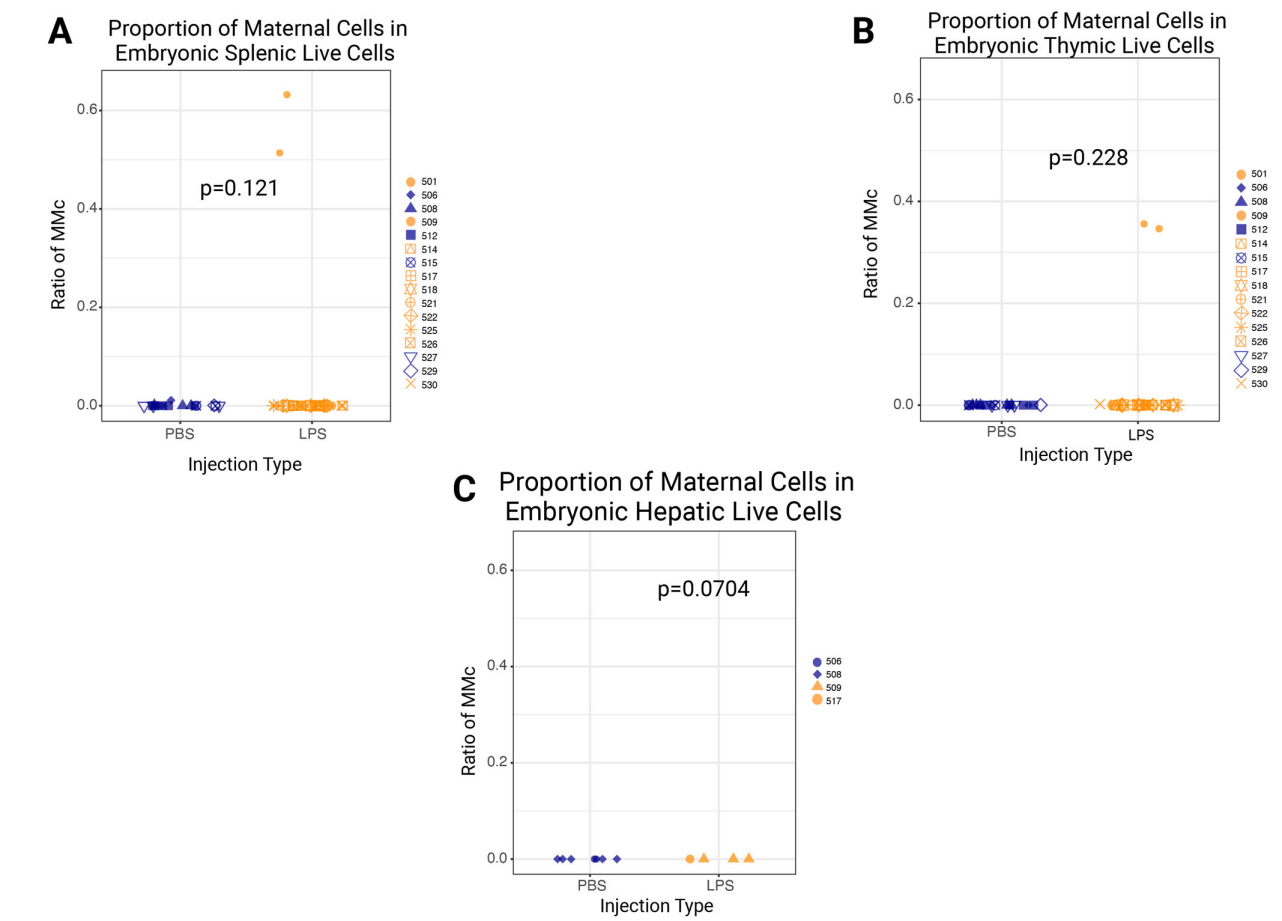
**No statistically significant difference was detected in the proportion of maternal cells between LPS-injected and PBS-injected control dams.** (A) Proportion of maternal cells in the seemingly healthy embryonic spleen. Embryos (*n*=27) from LPS-injected dams (*n*=10). Embryos (*n*=21) from PBS-injected dams (*n*=6). (B) Proportion of maternal cells in the seemingly healthy embryonic thymus. Embryos (*n*=27) from LPS-injected dams (*n*=10). Embryos (*n*=21) from PBS-injected dams (*n*=6). (C) Proportion of maternal cells in the seemingly healthy embryonic liver. Embryos (*n*=5) from LPS-injected dams (*n*=2). Embryos (*n*=7) from PBS-injected dams (*n*=2). All *P*-values were obtained by performing the Wilcoxon signed-rank test. All of the GFP+ (MMc) cell numbers are listed in Table S1.

To observe a potential change in the maternal cell proportion by the LPS injection, whole organ analysis was conducted on the embryonic murine spleen and thymus. Embryonic liver samples were diluted to around 10^6^ cells per well. FACS analysis showed no detectable variability in the proportion of GFP-positive cells among live spleens (*n*=47) (Fig. 4A), thymuses (*n*=47) (Fig. 4B), and livers (*n*=12) (Fig. 4C). The proportion of GFP-positive cells in the total live cell population of all the embryos derived from LPS-injected dams is spleen: 1.86% (38,266 cells); thymus: 11.3% (241,827 cells); and liver: <1% (29 cells). For embryos from PBS-injected dams, the proportions are spleen: <1% (349 cells); thymus: <1% (57 cells); and liver: <1% (four cells).

### No significant difference in MMc cell-type repertoire was detected

**Fig. 5.**
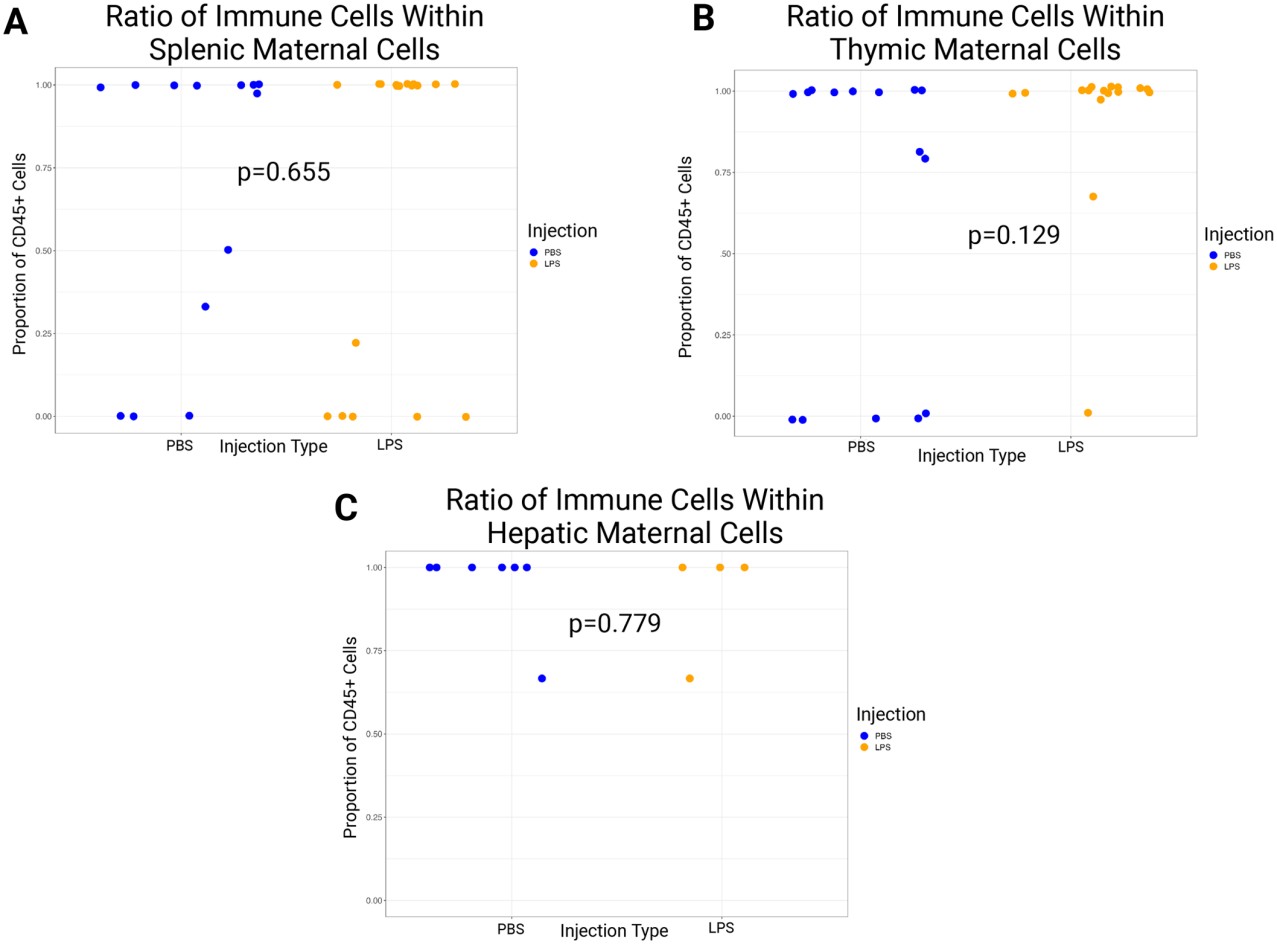
**No significant difference was detected in the proportion of immune maternal cells among maternal cells in LPS-injected and PBS-injected dams**. (A) Proportion of maternal cells in the seemingly healthy embryonic spleen. Embryos (*n*=27) from LPS-injected dams (*n*=10). Embryos (*n*=21) from PBS-injected dams (*n*=6). (B) Proportion of maternal cells in the seemingly healthy embryonic thymus. Embryos (*n*=27) from LPS-injected dams (*n*=10). Embryos (*n*=21) from PBS-injected dams (*n*=6). (C) Proportion of maternal cells in the seemingly healthy embryonic liver. Embryos (*n*=5) from LPS-injected dams (*n*=2). Embryos (*n*=7) from PBS-injected dams (*n*=2). All *P*-values were obtained by performing the Wilcoxon signed-rank test. All of the GFP+ (MMc) and CD45+ GFP+ cell numbers are listed in Table S1.

Previous studies of whole embryonic analysis suggested that, on average, the majority of the maternal cell population is immune-related (67%, 127 out of 191 cells), such as myeloid cells (36%, 69 out of 191 maternal cells) observed across 26 whole embryos (Fujimoto et al., 2022). The presence of immune-related maternal cells was categorized by their positive gating for CD45. The ratio represents the portion of CD45 among the live cells in the spleen (Fig. 5A), thymus (Fig. 5B), and liver (Fig. 5C). The proportion of immune-positive cells in the GFP-positive cell population for embryos from LPS-injected dams is spleen: 97.0% (37,122 cells); thymus: 99.4% (240,494 cells); and liver: 93.1% (27 cells). In the PBS-injected group, the spleen was 91.4% (319 cells); thymus: 85.9% (49 cells); and liver: 100% (four cells). Statistical comparison (Wilcoxon signed rank test) of PBS-injected and LPS-injected samples revealed that none of the organs showed statistical differences in the ratio of CD45 cells within the maternal cell population.

### Two littermates showed a high number of maternal cells

Two littermates from an LPS-injected dam showed a high frequency of maternal cells (Fig. S2). The maternal cell-type repertoire included CD45^+^ immune cells, CD19^+^ B cells, CD5^+^ T cells, CD11b^+^ myeloid cells, and Ly6b.2^+^ inflammatory monocytes and activated macrophages.

## DISCUSSION

In order to investigate potential novel biasing factors of MMc, we utilized mid-gestational MIA on the pregnant dam using LPS. It was a possibility that upregulation of immune cells from LPS-induced MIA would result in an influx of immune cells in progeny, with attempts to observe phenotypes with increased MMc previously untested. However, despite the embryonic phenotypes of the LPS-injected group, which suggests maternal immune activation, our FACS results suggest we were not able to detect a significant difference in the frequency or proportion of maternal cells in any organ in the embryos of seemingly healthy condition. Not every organ had maternal cells present (28/107 total organs), and organs with detectable maternal cells (79/107 total organs) showed them in relatively low frequency (Table S1, Figs S3 and S4). The inability to detect GFP-positive maternal cells in all embryos could result from the automatic electronic aborts during FACS that removes around 1 in 100 cell counts. Meanwhile, two embryos from the same LPS-injected dam (dam 501) appeared to have a seemingly higher frequency of maternal cells in their tested organs, the spleen and thymus. As the proportion of maternal cells has been found to naturally vary significantly even in non-transgenic embryos (Fujimoto et al., 2021), an increase of maternal cell frequency alone is insufficient to attribute to the response to inflammatory reactions. More samples are needed to determine if these samples are outliers due to technical errors or are naturally occurring, albeit rare, variations. Other MIA experiments utilize a higher dosage, risking higher mortality of both mother and pups (Fricke et al., 2018). One caveat of this study is that we did not monitor the level of immune response toward LPS. It is possible that survived embryos used as samples were those with a potentially weak reaction to LPS, and thus ended up showing a generally comparable number of MMc with that of PBS-injected. It would be worth designing this experimental component in future studies. Additionally, it is tempting to test if the embryos with lethal phenotypes (Fig. 3) produced by LPS injection had a higher frequency of MMc cells, as congenital diseases are often reported to have a higher frequency of MMc cells (Irie et al., 2009). The statistical independence of embryos is another factor that future studies should consider. In this study, we initially considered embryos from the same dam to be statistically independent of each other. However, a large MMc variance among dams (Kruskal–Wallis test, *P*=0.00019 for PBS group and *P*=3.09e-05 for LPS group) implied that this assumption may not stand. Although the results were essentially the same when average MMc frequencies of embryos from the same dam were used (Fig. S5), the fact that embryos from the same dam share the same maternal environment may warrant consideration.

The timing of murine immune system development may require additional consideration. Injection at 11.5 dpc was selected as it is right before the known migration of MMc at E12.5-E13, which precedes a high at parturition (Vernochet et al., 2007; Jeanty et al., 2014). However, landmark studies have determined key points of the murine immune system development that occur before the time of injection, where at E7 primitive hematopoiesis occurs in the yolk sac, followed by subsequent generation of hematopoietic stem cells (HSCs) resulting from endothelial-to-hematopoietic transition at E10.5 (Gao et al., 2018). Allowing the preliminary development of the embryonic immune system to occur before injection may have limited the full influential capacity of maternal cells. Implementing MIA before the known development of the spleen, liver, and thymus could provide a more explicit observation of the effect of MIA on the maternal cell frequency and cell-type repertoire without possibly clashing with the native fetal immune cell population. An attractive complementary approach to better investigate maternal immune cells would be to utilize CD45 congenic parents of the sampled embryos. Detecting the rare maternal cells in the progeny is met with several limitations, namely the need for a highly sensitive detection method and a high risk of contamination from the dam and other littermates. Another potential factor is a variable expression of GFP fluorescence, altered by autofluorescence or lack of expression. Although the detected cells may contain cells from GFP+ siblings as a result of mosaic embryos, it is unlikely that they biased the detected GFP+ cells. This is because the number ratio of GFP-positive maternal cells in the fetus did not change by the number, nor the ratio of GFP-positive siblings in the litters (Fujimoto et al., 2021). Murine organs are also fragile, where even slight mishandling may result in excess debris and uninterpretable analysis. The process from maternal dissection to cell sorting is extensive, taking several hours, which may affect the viability of cells.

As previously mentioned, variable frequencies of MMc have been reported among seemingly healthy littermates, implying the existence of an MMc biasing factor that influences the enigmatic cell inheritance. However, as we discovered, the frequency and cell-type repertoire remained stable even under immune-activated conditions, at least for viable embryos, implying the possible existence of a control system of MMc migration. This finding aligns with a recent breakthrough study comparing MMc following increased parity, which suggests a fixed niche of microchimeric cells (Shao et al., 2023). An unknown influence or control system derived from the mother or possibly even the fetus could be controlling the MMc frequency. With more experimentation, it would be interesting to explore other potential biasing factors of MMc, notably if implementing maternal stress or another form of MIA is sufficient, like the use of polyinosinic-polycytidylic acid (Poly I:C), which mimics viral infection. In parallel, implementing maternal immune suppression may be of interest, as maternal cells are proposed to be primarily immune-related and thus may result in a potential decrease of maternal cells in progeny. Further, activation or suppression of the fetal immune system may influence the maternal cell population, where different variations of the genetic condition severe combined immunodeficiency (SCID) showed both zero and total engraftment of circulating maternal T cells between ill infant patients (Müller et al., 2001). Apart from priming fetal tolerance, MMc further influences immune system development beyond parturition. Several emergent postnatal roles have recently been proposed for maternal cells (Borges et al., 2023). For example, the use of a low MMc model showed increased severity of neonatal infection (Stelzer et al., 2021). A recent human study revealed that unaffected infants born to HIV-positive mothers with fewer maternal cells showed a decreased T-cell response to vaccination at birth (Balle et al., 2022). Since we know maternal cells can last our whole lives, elucidating a functional purpose of MMc's variability would allow the utilization of its benefits while minimizing its detriments.

## MATERIALS AND METHODS

### Mouse breeding

All experimental procedures and mouse care were conducted under the guidelines and approval of the University of Tokyo (17-03). All mice were in a temperature-controlled environment with a 12-h light/dark cycle under *ad libitum* feeding. GFP heterozygous dams were acquired by mating ubiquitously expressing GFP (Okabe et al., 1997) male mice with wild-type BALB/cByJJcl female mice purchased from Clea Japan. Female virgin progeny from this mating scheme were subsequently mated with male wild-type BALB/cByJJcl mice purchased from Clea Japan. Pregnancy was confirmed following timed mating through plug confirmation, marking E0.5.

### MIA

MIA was implemented using LPS O111:B4 (Sigma-Aldrich, L2630). LPS was administered to pregnant dams at 11.5 dpc through intraperitoneal (i.p.) injections at a dosage of 75 µg/kg in sterile PBS.

### Organ dissection and preparation

Pregnant dams were euthanized at 18.5 dpc under anesthesia by cervical dislocation. The uterus was dissected and washed twice in PBS, with embryos subsequently separated individually in dishes containing ice-cold FACS buffer [1% g/ml BSA, 2 mM EDTA, and HBSS (−)]. The embryos were checked for GFP expression using UV detection. GFP-negative embryos were sorted for the extraction of the spleen, thymus, and liver. Immediately following dissection, organs were placed in ice-cold FACS buffer. Murine organs were mashed with a 70 µm filter, rinsed, and treated with 10X ACK lysis. Cell count estimation was completed using Countess II FL, with WT equipment controls and liver samples diluted to around 1×10^6^ cells. Samples were treated with antibodies and sorted into sterile 96-well plates. Fluorescence-activated cell sorting was conducted using BD FACSAria III maintained by the OneStop Facility, University of Tokyo.

### Antibody panel

A multicolor antibody was implemented on all murine embryonic organs. The panel includes Invitrogen LIVE/DEAD Fixable Near-IR Dead Cell Stain Kit (L34975), CD45 (Miltenyi Biotec order no. 130-123-879), CD5 (BD Biosciences, cat. no. 562739), CD19 (BD Biosciences, cat. no. 562956), CD11b, (BD Biosciences, cat. no. 561689), Ly6b (Miltenyi Biotec order no. 130-102-322).

### Statistical testing

Alpha level of 0.01 was used for statistical significance throughout the analyses.

## Supplementary Material

10.1242/biolopen.061830_sup1 Supplementary information

Table S1. Total Cell Counts of Flow Cytometric Analysis.

## References

[BIO061830C1] Aydın, M. Ş., Yiğit, E. N., Vatandaşlar, E., Erdoğan, E. and Öztürk, G. (2018). Transfer and integration of breast milk stem cells to the brain of suckling pups. *Sci. Rep.* 8, 14289. 10.1038/s41598-018-32715-5 30250150 PMC6155265

[BIO061830C2] Bakkour, S., Baker, C. A. R., Tarantal, A. F., Wen, L., Busch, M. P., Lee, T. H. and Mccune, J. M. (2014). Analysis of maternal microchimerism in rhesus monkeys (Macaca mulatta) using real-time quantitative PCR amplification of MHC polymorphisms. *Chimerism* 5, 6-15. 10.4161/chim.27778 24451553 PMC3988117

[BIO061830C3] Balle, C., Armistead, B., Kiravu, A., Song, X., Happel, A.-U., Hoffmann, A. A., Kanaan, S. B., Nelson, J. L., Gray, C. M., Jaspan, H. B. et al. (2022). Factors influencing maternal microchimerism throughout infancy and its impact on infant T cell immunity. *J. Clin. Investig.* 132, e148826. 10.1172/jci148826 35550376 PMC9246390

[BIO061830C4] Borges, A., Castellan, F. and Irie, N. (2023). Emergent roles of maternal microchimerism in postnatal development. *Dev. Growth Diff.* 65, 75-81. 10.1111/dgd.12830 36519824

[BIO061830C5] Castellan, F. S. and Irie, N. (2022). Postnatal depletion of maternal cells biases T lymphocytes and natural killer cells’ profiles toward early activation in the spleen. *Biol. Open* 11, bio059334. 10.1242/bio.059334 36349799 PMC9672855

[BIO061830C6] Dutta, P. and Burlingham, W. J. (2011). Microchimerism: tolerance vs. sensitization. *Curr. Opin. Organ Transplantat.* 16, 359-365. 10.1097/MOT.0b013e3283484b57 PMC3337767 21666480

[BIO061830C7] Dutta, P., Molitor-Dart, M., Bobadilla, J. L., Roenneburg, D. A., Yan, Z., Torrealba, J. R. and Burlingham, W. J. (2009). Microchimerism is strongly correlated with tolerance to noninherited maternal antigens in mice. *Blood* 114, 3578-3587. 10.1182/blood-2009-03-213561 19700665 PMC2766676

[BIO061830C8] Fricke, E. M., Elgin, T. G., Gong, H., Reese, J., Gibson-Corley, K. N., Weiss, R. M., Zimmerman, K., Bowdler, N. C., Kalantera, K. M., Mills, D. A. et al. (2018). Lipopolysaccharide-induced maternal inflammation induces direct placental injury without alteration in placental blood flow and induces a secondary fetal intestinal injury that persists into adulthood. *Am. J. Reprod. Immunol.* 79, e12816. 10.1111/aji.12816 29369434 PMC5908742

[BIO061830C9] Fujimoto, K., Nakajima, A., Hori, S. and Irie, N. (2021). Whole embryonic detection of maternal microchimeric cells highlights significant differences in their numbers among individuals. *PLoS ONE* 16, e12816. 10.1371/journal.pone.0261357 PMC8699925 34941916

[BIO061830C10] Fujimoto, K., Nakajima, A., Hori, S., Tanaka, Y., Shirasaki, Y., Uemura, S. and Irie, N. (2022). Whole-embryonic identification of maternal microchimeric cell types in mouse using single-cell RNA sequencing. *Sci. Rep.* 12, 18313. 10.1038/s41598-022-20781-9 36333354 PMC9636240

[BIO061830C11] Gammill, H. S. and Nelson, J. L. (2010). Naturally acquired microchimerism. *Int. J. Dev. Biol.* 54, 531-543. 10.1387/ijdb.082767hg 19924635 PMC2887685

[BIO061830C12] Gao, X., Xu, C., Asada, N. and Frenette, P. S. (2018). The hematopoietic stem cell niche: from embryo to adult. *Development* 145, dev139691. 10.1242/dev.139691 29358215 PMC5825844

[BIO061830C13] Irie, N., Muraji, T., Hosaka, N., Takada, Y., Sakamoto, S. and Tanaka, K. (2009). Maternal HLA class I compatibility in patients with biliary atresia. *J. Pediatr. Gastroenterol. Nutr.* 49, 488-492. 10.1097/MPG.0b013e31819a4e2c 19590453

[BIO061830C14] Jeanty, C., Derderian, S. C. and Mackenzie, T. C. (2014). Maternal-fetal cellular trafficking: clinical implications and consequences. *Curr. Opin. Pediatr.* 26, 377-382. 10.1097/MOP.0000000000000087 24759226 PMC4079801

[BIO061830C15] Jonsson, A. M., Uzunel, M., Götherström, C., Papadogiannakis, N. and Westgren, M. (2008). Maternal microchimerism in human fetal tissues. *Am. J. Obstet. Gynecol.* 198, 325.e1-325.e6. 10.1016/j.ajog.2007.09.047 18191801

[BIO061830C16] Kinder, J. M., Jiang, T. T., Ertelt, J. M., Xin, L., Strong, B. S., Shaaban, A. F. and Way, S. S. (2015). Cross-generational reproductive fitness enforced by microchimeric maternal cells. *Cell* 162, 505-515. 10.1016/j.cell.2015.07.006 26213383 PMC4522363

[BIO061830C17] Lambert, N. C., Erickson, T. D., Yan, Z., Pang, J. M., Guthrie, K. A., Furst, D. E. and Nelson, J. L. (2004). Quantification of maternal microchimerism by HLA-specific real-time polymerase chain reaction: studies of healthy women and women with scleroderma. *Arthritis Rheum.* 50, 906-914. 10.1002/art.20200 15022334

[BIO061830C18] Lo, Y. M., Lo, E. S., Watson, N., Noakes, L., Sargent, I. L., Thilaganathan, B. and Wainscoat, J. S. (1996). Two-way cell traffic between mother and fetus: biologic and clinical implications. *Blood* 88, 4390-4395. 10.1182/blood.V88.11.4390.bloodjournal88114390 8943877

[BIO061830C19] Loubière, L. S., Lambert, N. C., Flinn, L. J., Erickson, T. D., Yan, Z., Guthrie, K. A., Vickers, K. T. and Nelson, J. L. (2006). Maternal microchimerism in healthy adults in lymphocytes, monocyte/macrophages and NK cells. *Lab. Investig.* 86, 1185-1192. 10.1038/labinvest.3700471 16969370

[BIO061830C20] Mishra, A., Lai, G. C., Yao, L. J., Aung, T. T., Shental, N., Rotter-Maskowitz, A., Shepherdson, E., Singh, G. S. N., Pai, R., Shanti, A. et al. (2021). Microbial exposure during early human development primes fetal immune cells. *Cell* 184, 3394-3409.e20. 10.1016/j.cell.2021.04.039 34077752 PMC8240556

[BIO061830C21] Mold, J. E., Michaëlsson, J., Burt, T. D., Muench, M. O., Beckerman, K. P., Busch, M. P., Lee, T. H., Nixon, D. F. and Mccune, J. M. (2008). Maternal alloantigens promote the development of tolerogenic fetal regulatory T cells in utero. *Science* 322, 1562-1565. 10.1126/science.1164511 19056990 PMC2648820

[BIO061830C22] Müller, S. M., Ege, M., Pottharst, A., Schulz, A. S., Schwarz, K. and Friedrich, W. (2001). Transplacentally acquired maternal T lymphocytes in severe combined immunodeficiency: a study of 121 patients. *Blood* 98, 1847-1851. http://ashpublications.org/blood/article-pdf/98/6/1847/1677058/h8180101847.pdf. 10.1182/blood.V98.6.1847 11535520

[BIO061830C23] Muraji, T., Hosaka, N., Irie, N., Yoshida, M., Imai, Y., Tanaka, K., Takada, Y., Sakamoto, S., Haga, H. and Ikehara, S. (2008). Maternal microchimerism in undrlying pathogenesis of biliary atresia: quantification and phenotypes of maternal cells in the liver. *Pediatrics* 121, 517-521. 10.1542/peds.2007-0568 18310200

[BIO061830C24] Muraji, T., Suskind, D. L. and Irie, N. (2009). Biliary atresia: a new immunological insight into etiopathogenesis. *Expert Rev. Gastroenterol. Hepatol.* 3, 599-606. 10.1586/egh.09.61 19929581

[BIO061830C25] Nelson, J. L. (2012). The otherness of self: microchimerism in health and disease. *Trends Immunol.* 33, 421-427. 10.1016/j.it.2012.03.002 22609148 PMC3516290

[BIO061830C26] Nelson, J. L., Gillespie, K. M., Lambert, N. C., Stevens, A. M., Loubiere, L. S., Rutledge, J. C., Leisenring, W. M., Erickson, T. D., Yan, Z., Mullarkey, M. E. et al. (2007). Maternal microchimerism in peripheral blood in type 1 diabetes and pancreatic islet β cell microchimerism. *Proc. Natl Acad. Sci. USA* 104, 1637-1642. 10.1073/pnas.0606169104 17244711 PMC1785262

[BIO061830C27] Okabe, M., Ikawa, M., Kominami, K., Nakanishi, T. and Nishimune, Y. (1997). “Green mice” as a source of ubiquitous green cells. *FEBS Lett.* 407, 313-319. 10.1016/S0014-5793(97)00313-X 9175875

[BIO061830C38] Piotrowski, P., Croy, B. A. (1996). Maternal cells are widely distributed in murine fetuses in utero. *Biology of Reproduction*. 54, 1103-1110. 10.1095/biolreprod54.5.1103 8722632

[BIO061830C28] Reed, A. M., Mcnallan, K., Wettstein, P., Vehe, R. and Ober, C. (2004). Does HLA-dependent chimerism underlie the pathogenesis of juvenile dermatomyositis? *J. Immunol.* 172, 5041-5046. 10.4049/jimmunol.172.8.5041 15067086

[BIO061830C29] Roy, E., Leduc, M., Guegan, S., Rachdi, L., Kluger, N., Scharfmann, R., Aractingi, S. and Khosrotehrani, K. (2011). Specific maternal microchimeric T cells targeting fetal antigens in β cells predispose to auto-immune diabetes in the child. *J. Autoimmun.* 36, 253-262. 10.1016/j.jaut.2011.02.003 21414756

[BIO061830C30] Shao, T.-Y., Kinder, J. M., Harper, G., Pham, G., Peng, Y., Liu, J., Gregory, E. J., Sherman, B. E., Wu, Y., Iten, A. E. et al. (2023). Reproductive outcomes after pregnancy-induced displacement of preexisting microchimeric cells. *Science* 381, 1324-1330. 10.1126/science.adf9325 37733857 PMC10877202

[BIO061830C31] Shimizu, Y., Sakata-Haga, H., Saikawa, Y. and Hatta, T. (2023). Influence of immune system abnormalities caused by maternal immune activation in the postnatal period. *Cells* 12, 741. 10.3390/cells12050741 36899877 PMC10001371

[BIO061830C32] Silveira, T. R., Salzano, F. M., Donaldson, P. T., Mieli-Vergani, G., Howard, E. R. and Mowat, A. P. (1993). Association between HLA and Extrahepatic Biliary Atresia. *J. Pediatr. Gastroenterol. Nutr.* 16, 114-117. 10.1097/00005176-199302000-00002 8450374

[BIO061830C33] Snethen, H., Ye, J., Gillespie, K. M. and Scolding, N. J. (2020). Maternal micro-chimeric cells in the multiple sclerosis brain. *Mult. Scler. Relat. Disord.* 40, 101925. 10.1016/j.msard.2020.101925 31986425

[BIO061830C34] Stelzer, I. A., Urbschat, C., Schepanski, S., Thiele, K., Triviai, I., Wieczorek, A., Alawi, M., Ohnezeit, D., Kottlau, J., Huang, J. et al. (2021). Vertically transferred maternal immune cells promote neonatal immunity against early life infections. *Nat. Commun.* 12, 4706. 10.1038/s41467-021-24719-z 34349112 PMC8338998

[BIO061830C35] Stevens, A. M., Hermes, H. M., Rutledge, J. C., Buyon, J. P. and Nelson, J. L. (2003). Myocardial-tissue-specific phenotype of maternal microchimerism in neonatal lupus congenital heart block. *Lancet* 362, 1617-1623. 10.1016/S0140-6736(03)14795-2 14630442

[BIO061830C36] Stevens, A. M., Hermes, H. M., Kiefer, M. M., Rutledge, J. C. and Nelson, J. L. (2009). Chimeric maternal cells with tissue-specific antigen expression and morphology are common in infant tissues. *Pediatr. Dev. Pathol.* 12, 337-346. 10.2350/08-07-0499.1 18939886 PMC2783488

[BIO061830C37] Vernochet, C., Caucheteux, S. M. and Kanellopoulos-Langevin, C. (2007). Bi-directional cell trafficking between mother and fetus in mouse placenta. *Placenta* 28, 639-649. 10.1016/j.placenta.2006.10.006 17116327

