# Supplementary material for: Maternal immune activation does not affect maternal microchimeric cells
Source: Biol Open. 2024 Dec 23;13(12):bio061830. doi: 10.1242/bio.061830 (PMC11695574; doi:10.1242/bio.061830)
Supplement: Supplementary information [file biolopen-13-061830-s1.pdf]

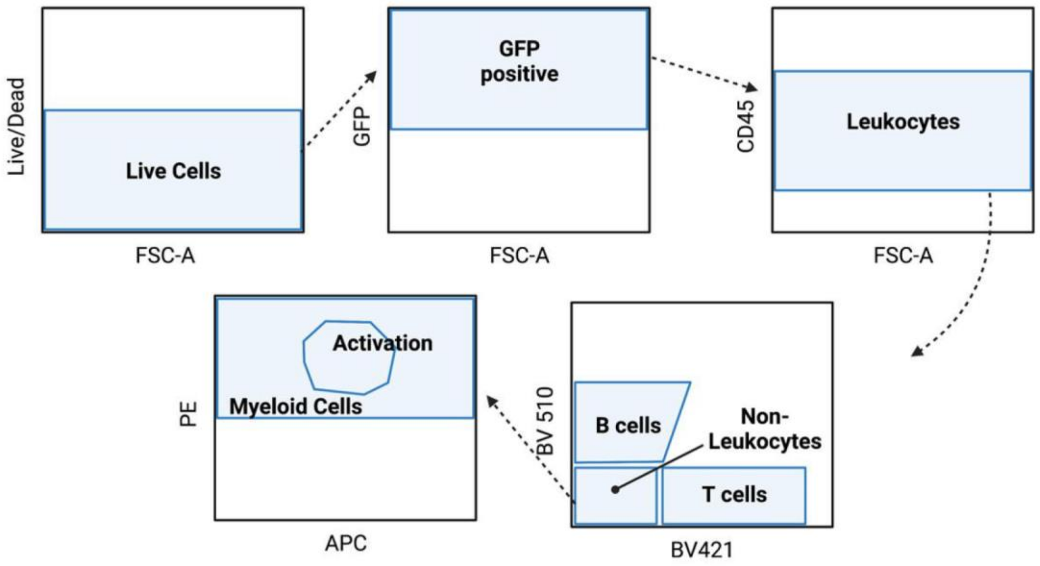

Fig. S1. Gating schematic for analyzing GFP expressing maternal cells in embryonic organs. Treated samples are gated for viability and then for GFP fluorescence. Cells in the GFP-positive gate are then gated for different immune cell classifications, including B cells and T cells, or classified as myeloid cells by gating against leukocyte markers. Myeloid cells are gated further with activated/inflammation markers.

Biology Open • Supplementary information

A Dam 501: Embryo 1 FACS

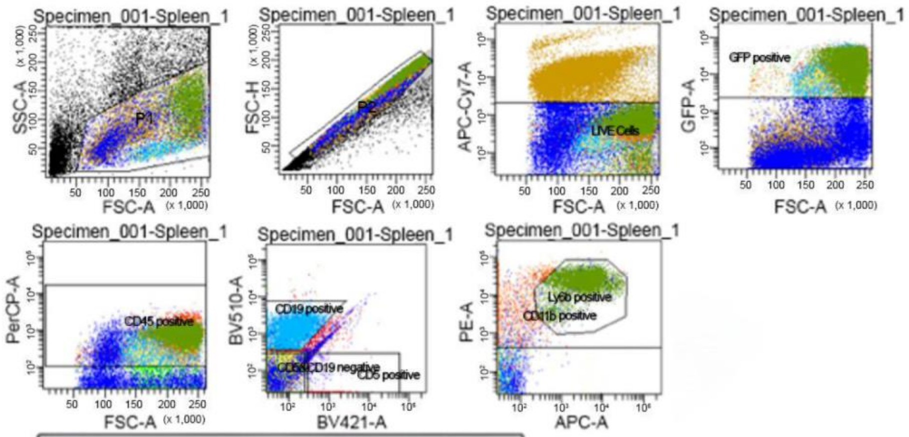

Embryo 1 Spleen

Population	#Events	%Parent	%Total
All Events	194,515	####	100.0
P1	98,968	50.9	50.9
P2	73,570	74.3	37.8
LIVE Cells	44,984	61.1	23.1
GFP positive	28,542	63.4	14.7
CD45 positive	27,670	96.9	14.2
CD19 positive	7,276	26.3	3.7
CD5 positive	50	0.2	0.0
CD5&CD19 neg.	19,462	70.3	10.0
CD11b pos.	18,310	94.1	9.4
Ly6b.2 pos.	16,027	87.5	8.2

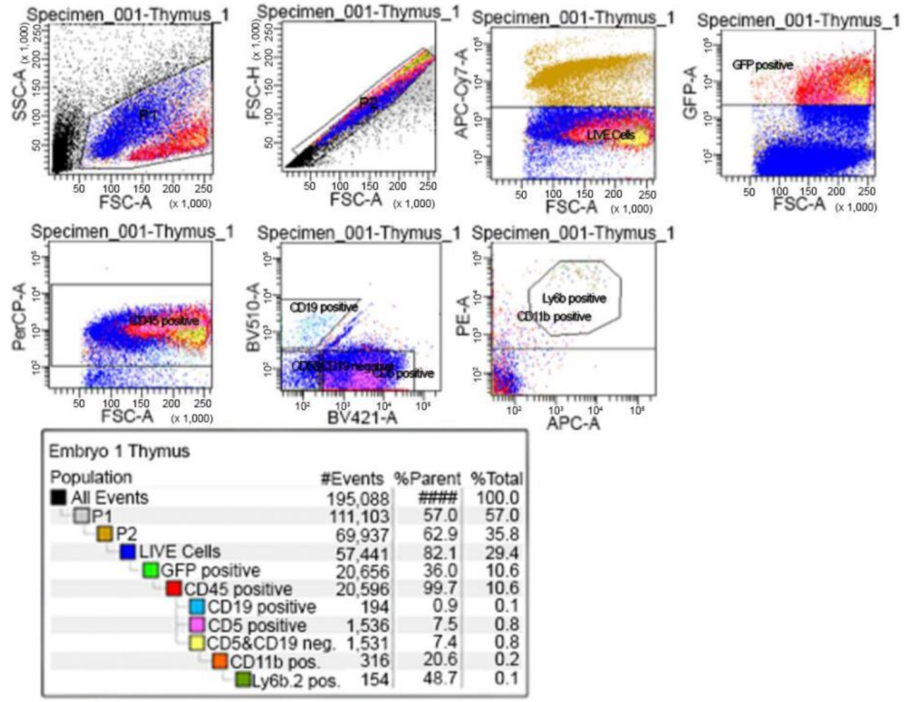

Embryo 1 Thymus

Population	#Events	%Parent	%Total
All Events	195,088	####	100.0
P1	111,103	57.0	57.0
P2	69,937	62.9	35.8
LIVE Cells	57,441	82.1	29.4
GFP positive	20,656	36.0	10.6
CD45 positive	20,596	99.7	10.6
CD19 positive	194	0.9	0.1
CD5 positive	1,536	7.5	0.8
CD5&CD19 neg.	1,531	7.4	0.8
CD11b pos.	316	20.6	0.2
Ly6b.2 pos.	154	48.7	0.1

Biology Open • Supplementary information

B Dam 501: Embryo 2 FACS

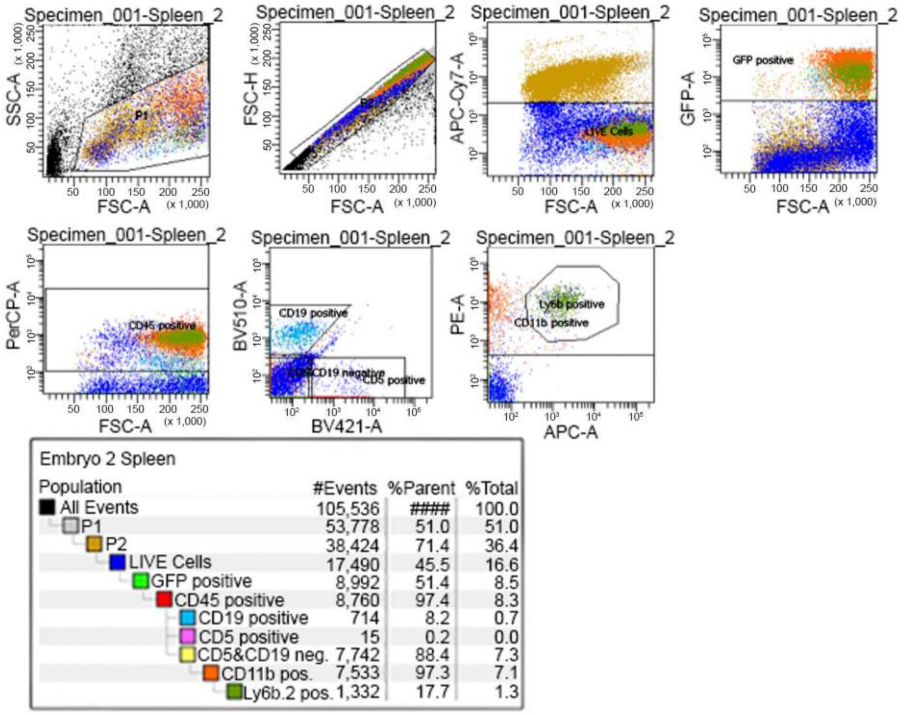

Embryo 2 Spleen

Population	#Events	%Parent	%Total
All Events	105,536	####	100.0
P1	53,778	51.0	51.0
P2	38,424	71.4	36.4
LIVE Cells	17,490	45.5	16.6
GFP positive	8,992	51.4	8.5
CD45 positive	8,760	97.4	8.3
CD19 positive	714	8.2	0.7
CD5 positive	15	0.2	0.0
CD5&CD19 neg.	7,742	88.4	7.3
CD11b pos.	7,533	97.3	7.1
Ly6b.2 pos.	1,332	17.7	1.3

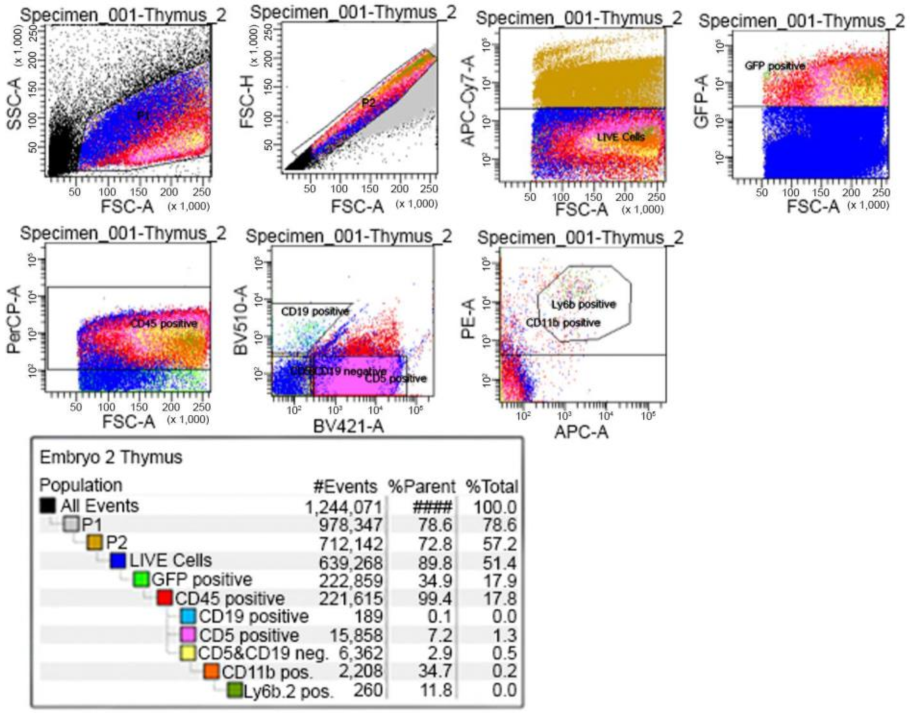

Embryo 2 Thymus

Population	#Events	%Parent	%Total
All Events	1,244,071	####	100.0
P1	978,347	78.6	78.6
P2	712,142	72.8	57.2
LIVE Cells	639,268	89.8	51.4
GFP positive	222,859	34.9	17.9
CD45 positive	221,615	99.4	17.8
CD19 positive	189	0.1	0.0
CD5 positive	15,858	7.2	1.3
CD5&CD19 neg.	6,362	2.9	0.5
CD11b pos.	2,208	34.7	0.2
Ly6b.2 pos.	260	11.8	0.0

Fig. S2. Two embryos showed a high number of GFP-positive maternal cells in both the spleen and thymus. A. Embryo 1 FACS showed that maternal cells comprised 63.4% of the splenic cell population and 36% of the thymic cell population. B. Embryo 2 FACS showed that maternal cells comprised 51.4% of the splenic cell population and 34.9% of the thymic cell population.

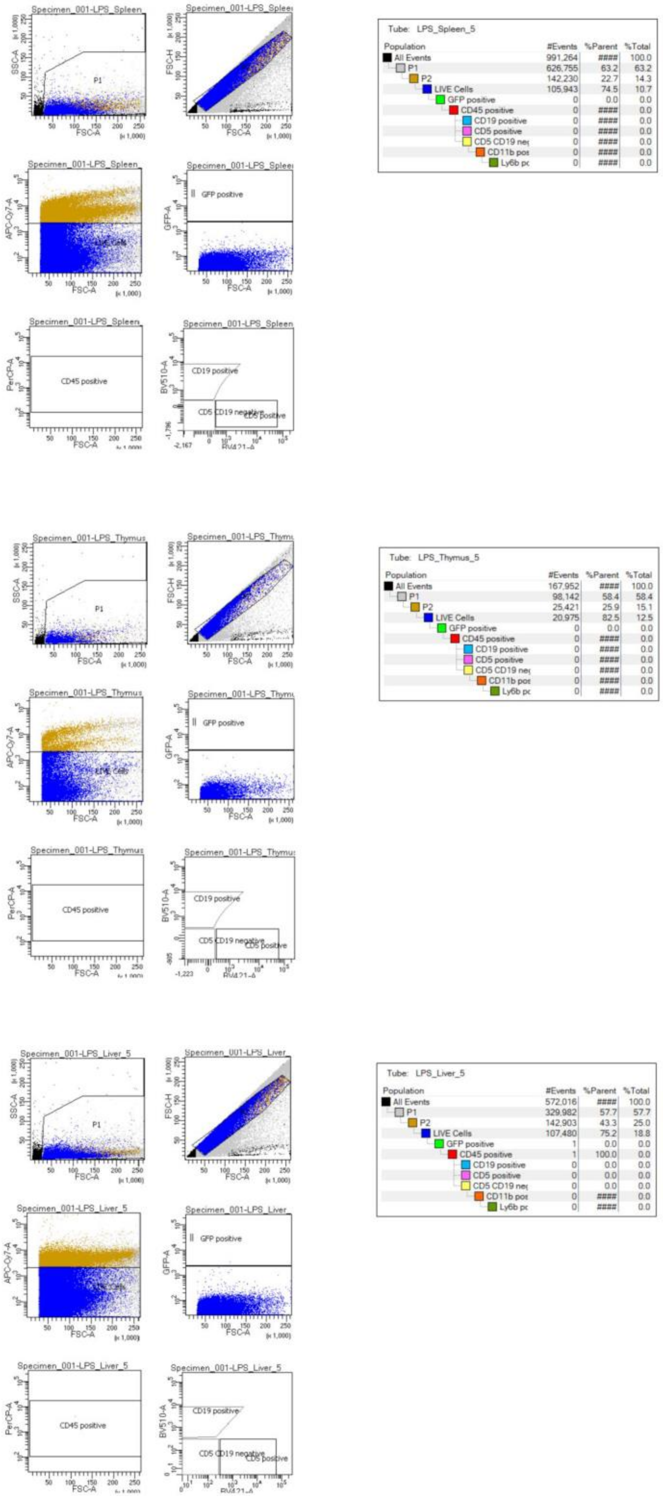

Fig. S3. Example of Low/Undetectable GFP positive cells in FACS Analysis Spleen, thymus, and liver analysis of LPS-injected dam (dam 509, embryo 5) and PBS-injected dam (dam 506, embryo 6).

Biology Open • Supplementary information

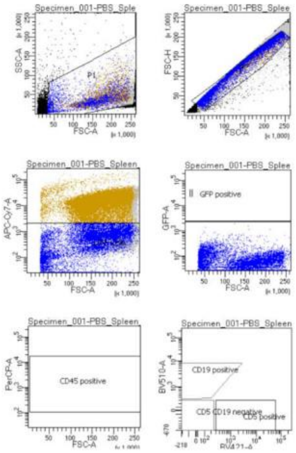

Tube: PBS_Spleen_6

Population	#Events	%Parent	%Total
All Events	188,384	####	100.0
P1	58,452	31.0	31.0
P2	36,253	62.0	19.2
LIVE Cells	12,392	34.2	6.6
GFP positive	0	0.0	0.0
CD45 positive	0	####	0.0
CD19 positive	0	####	0.0
CD5 positive	0	####	0.0
CD5 CD19 ne(	0	####	0.0
CD11b pos	0	####	0.0
Ly6b pc	0	####	0.0

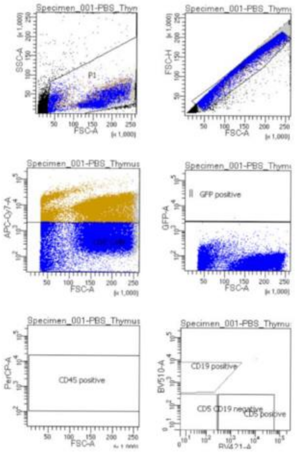

Tube: PBS_Thymus_6

Population	#Events	%Parent	%Total
All Events	175,008	####	100.0
P1	59,883	34.2	34.2
P2	47,111	78.7	26.9
LIVE Cells	24,387	51.8	13.9
GFP positive	1	0.0	0.0
CD45 positive	1	100.0	0.0
CD19 positive	0	0.0	0.0
CD5 positive	0	0.0	0.0
CD5 CD19 ne(	0	0.0	0.0
CD11b pos	0	####	0.0
Ly6b pc	0	####	0.0

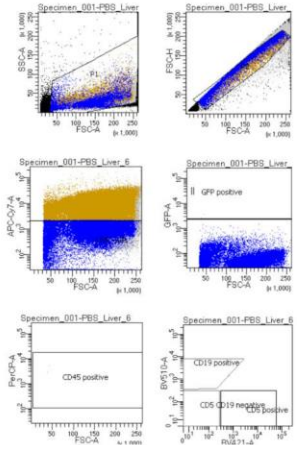

Tube: PBS_Liver_6

Population	#Events	%Parent	%Total
All Events	305,296	####	100.0
P1	142,456	46.7	46.7
P2	115,739	81.2	37.9
LIVE Cells	30,078	26.0	9.9
GFP positive	2	0.0	0.0
CD45 positive	2	100.0	0.0
CD19 positive	2	100.0	0.0
CD5 positive	0	0.0	0.0
CD5 CD19 ne(	0	0.0	0.0
CD11b pos	0	####	0.0
Ly6b pc	0	####	0.0

Fig. S4. Example of Low/Undetectable GFP positive cells in FACS Analysis Spleen, thymus, and liver analysis of LPS-injected dam (dam 509, embryo 5) and PBS-injected dam (dam 506, embryo 6).

Biology Open • Supplementary information

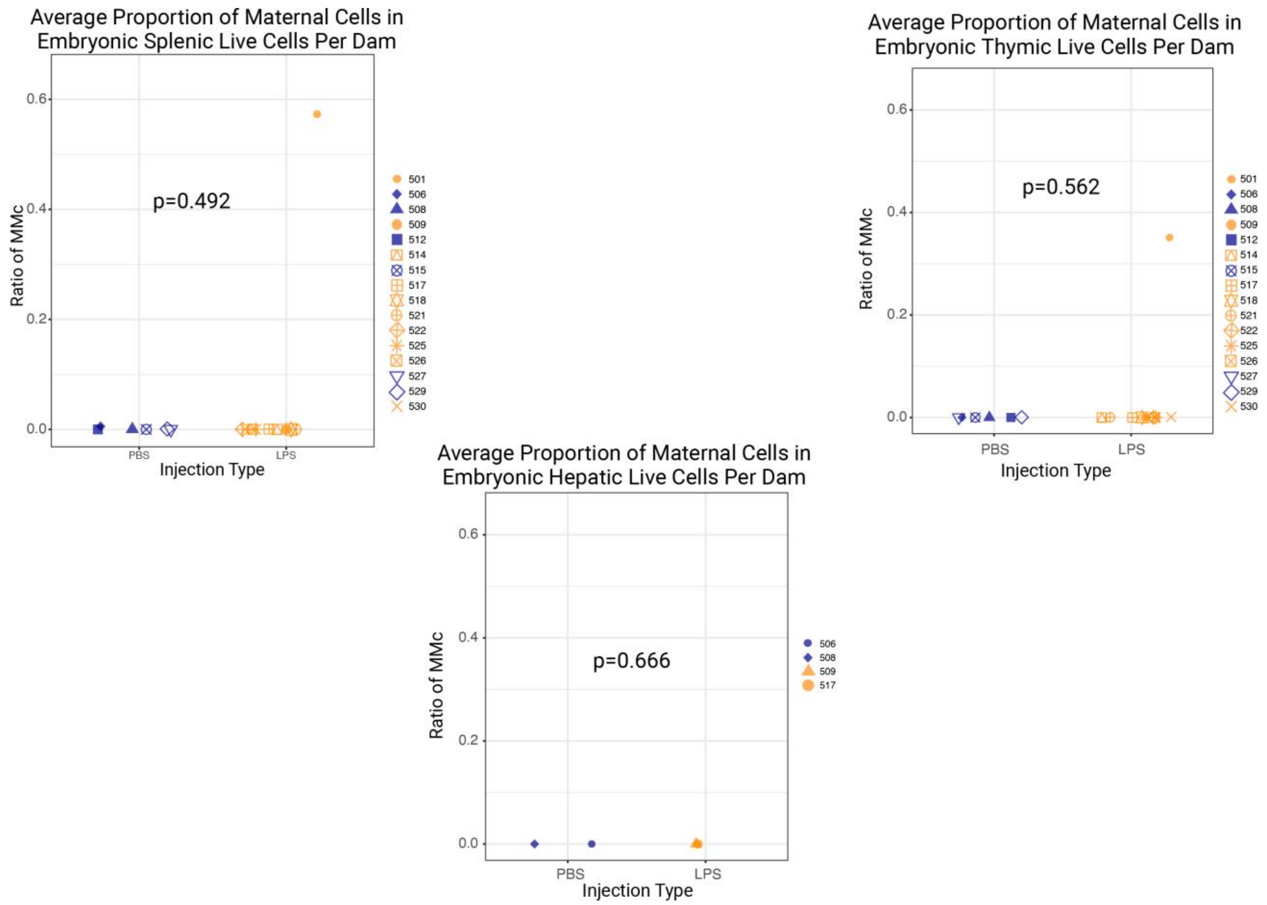

Fig. S5. Average MMc Ratio per Dam. A. Average proportion of maternal cells in the seemingly healthy embryonic spleen. LPS-injected dams (n=10). PBS-injected dams (n=6). B. Average proportion of maternal cells in the seemingly healthy embryonic thymus. LPS-injected dams (n=10). PBS-injected dams (n=6) C. Average proportion of maternal cells in the seemingly healthy embryonic liver. LPS-injected dams (n=2). PBS-injected dams (n=2). All P values were obtained by performing the Wilcoxon signed-rank test.

Table S1. Total Cell Counts of Flow Cytometric Analysis.

Available for download at
https://journals.biologists.com/bio/article-lookup/doi/10.1242/bio.061830#supplementary-data
